# Pheochromocytoma and multicentric giant cell tumor of bone

**DOI:** 10.20945/2359-4292-2026-0065

**Published:** 2026-05-01

**Authors:** João Vicente Silveira, Madson Q. Almeida, Olavo Pires de Camargo, Julio Cesar Carvalho Nardelli, Andre Ferrari de Franca Camargo, Henrique Barbosa Ribeiro, Luiz Aparecido Bortolotto, Heno Ferreira Lopes, Fernanda M. Consolim-Colombo

**Affiliations:** 1 Instituto do Coração do Hospital das Clínicas da Faculdade de Medicina da Universidade de São Paulo, São Paulo, SP, Brasil; 2 Unidade de Suprarrenal, Endocrinologia do Desenvolvimento, Laboratório de Hormônios e Genética Molecular - LIM42, Divisão de Endocrinologia e Metabologia, Hospital das Clínicas, Faculdade de Medicina da Universidade de São Paulo, São Paulo, SP, Brasil; 3 Instituto de Ortopedia e Traumatologia do Hospital das Clínicas da Faculdade de Medicina da Universidade de São Paulo, São Paulo, SP, Brasil; 4 Universidade Nove de Julho, São Paulo, SP, Brasil

**Keywords:** Pheochromocytoma, paragangliomas, giant cell bone tumors, hypertension, genetic

## Abstract

Pheochromocytomasand paragangliomas (PPGLs) are rare catecholamine-secreting tumors that can affect systemic physiology, including bone metabolism. Although they are typically associated with genetic syndromes such as MEN2 and von Hippel-Lindau disease, PPGLs are not commonly linked to primary bone tumors. However, recent findings suggest a novel cancer syndrome involving both PPGLs and giant cell tumor of bone (GCTB) through H3F3A mutations. We report a rare case of a 53-year-old woman with multicentric GCTB, initially diagnosed at age 24, who subsequently developed bilateral pheochromocytomas. Following multiple recurrences of skeletal tumors and eventual leg amputation, imaging and biochemical evaluation revealed bilateral adrenal tumors with markedly elevated catecholamine levels. Genetic analysis identified mosaicism for a pathogenic H3F3A (G35W) variant in both adrenal and bone tumors. Surgical resection of both adrenal glands normalized catecholamine levels and resolved hypertension. The patient has remained tumor-free and normotensive for five years following adrenalectomy. This case supports the existence of a shared pathogenic mechanism linking PPGLs and GCTBs, likely mediated by postzygotic H3F3A mutations. Recognition of this association is crucial for early diagnosis, genetic counseling, and management of similar cases.

## INTRODUCTION

Pheochromocytomas and paragangliomas (PPGLs) are rare tumors arising from neural crest cells and are characterized by catecholamine secretion. These tumors frequently exhibit genetic mutations affecting various biological pathways, notably influencing bone metabolism via catecholamine excess, which increases bone resorption and leads to secondary osteoporosis. Although PPGLs are commonly associated with genetic syndromes such as Multiple Endocrine Neoplasia type 2 (MEN2) and von Hippel-Lindau disease ^(1)^, pheochromocytomas are not typically linked with primary bone tumors. However, a prior publication identified a novel cancer syndrome connecting PPGLs with giant cell tumors of bone (GCT) through specific genetic mutations ^(2)^. Additionally, case reports in the literature have described occurrences of PPGLs coexisting with giant cell bone tumors. These discoveries highlight the need for further investigation into possible shared origins of these tumors, emphasizing the importance of understanding genetic risk in affected individuals.

## CASE PRESENTATION

We present the case of a 53-year-old woman who was initially diagnosed with a giant cell tumor of bone in the right tibia in 1995 at the age of 24. During follow-up, the tumor spread to various segments of her skeleton. In 2008, she began attending the outpatient clinic of the Orthopedic Unit at the Clinical Hospital, with a diagnosis of multicentric giant cell tumor of bone, as confirmed by a 2015 scintigraphy examination and biopsy fragments, which revealed a giant cell tumor of bone with areas of necrosis and a fibrocystic pattern. Due to multiple recurrences of the tumor in the right tibia, she underwent several resections, ultimately leading to a leg amputation in 2018, at the age of 47. Additionally, during a chest and lung tomography performed in the same period, two nodular images in the region of the adrenal glands were detected, measuring approximately 5.9 cm on the left and 4.5 cm on the right. These findings were considered indeterminate in this non-contrast study and were not further investigated at that time.

In 2019, on day 1, the patient began reporting episodes of headache, palpitations, and sweating, which had begun six months prior. She was diagnosed with hypertension at another clinic and was treated with losartan 50 mg twice daily, which reduced but did not normalize her blood pressure (140/90 mmHg). Her headache episodes became more frequent and were associated with thoracic discomfort.

On day 2, the patient presented to the emergency room with a hypertensive emergency (BP 240/ 120 mmHg), complicated by acute pulmonary edema and myocardial injury, following the sudden onset of severe dyspnea and chest pain during physiotherapy. After stabilization on day 3, cardiac catheterization confirmed a type II myocardial infarction, excluding coronary and renal artery obstruction. However, findings of atypical adrenal vasculature prompted bilateral adrenal angiography, which revealed large masses in both adrenal glands (**Figures 1A-1B**).

**Figure 1:**
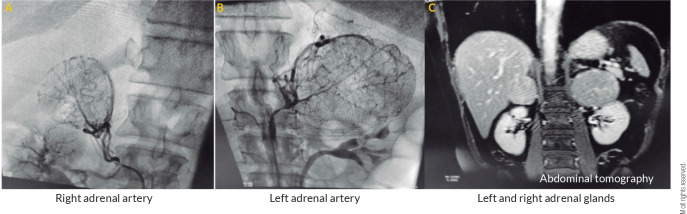
Adrenal artery catheterization (A and B) and abdominal CT scan (C). **A** and **B**) Injection of contrast into the adrenal arteries highlighting intense vascularization in the regions of the right and left adrenal glands, indicating the presence of bilateral tumor mass; **C**) Abdominal CT scan evidencing large tumor masses in both adrenal glands.

Subsequently, a CT scan of the abdomen was performed, revealing masses in the adrenal regions (40 Hounsfield units), measuring 10 cm in the left adrenal gland and 8 cm in the right adrenal gland. Both masses were compressing the kidneys. In this context, the possibility of pheochromocytoma was appropriately considered (**Figure 1C**). The suspicion of pheochromocytoma prompted confirmatory laboratory and specific imaging tests. The results of the confirmatory laboratory tests are presented in **Table 1**. Positron emission tomography/magnetic resonance imaging (PET/MRI) with somatostatin analog (68Ga-DOTATATE) demonstrated globular lesions in the adrenal glands with heterogeneous radiopharmaceutical uptake, well-defined margins, and regular contours. The left adrenal lesion exhibited a markedly higher concentration of radiopharmaceutical, measuring 8.0 × 6.2 × 5.5 cm (AP × CC × LL) and displaced the tail of the pancreas anteriorly. The right adrenal lesion showed a moderate concentration of radiopharmaceutical and measured 5.4 × 5.2 × 4.3 cm (AP × CC × LL), maintaining extensive contact with the inferior vena cava. Additionally, a focal area of discrete anomalous radiopharmaceutical accumulation was observed in the intramedullary region of the left distal radius (**Figure 2**).

**Table 1:** Plasma and urine catecholamine, plasma and urine metanephrine measurements pre-adrenalectomy

Variables	Values	Reference values
Plasma norepinephrine (pg/mL)	15,809	70-750 pg/mL
Plasma epinephrine (pg/mL)	Undetectable	0-110 pg/mL
Plasma dopamine (pg/mL)	Undetectable	Undetectable
Plasma metanephrine (nmol/L)	4.1	< 0.5 nmol/L
Urine norepinephrine (µg/24 h)	1,292	14-80 µg/24 h
Urine epinephrine (µg/24 h)	68	0.5-20 µg/24 h
Urine dopamine (µg/24 h)	1,536	65-400 µg/24 h
Urine metanephrine (µg/24 h)	2.5	0.05-1.2 µg/24 h

**Figure 2:**
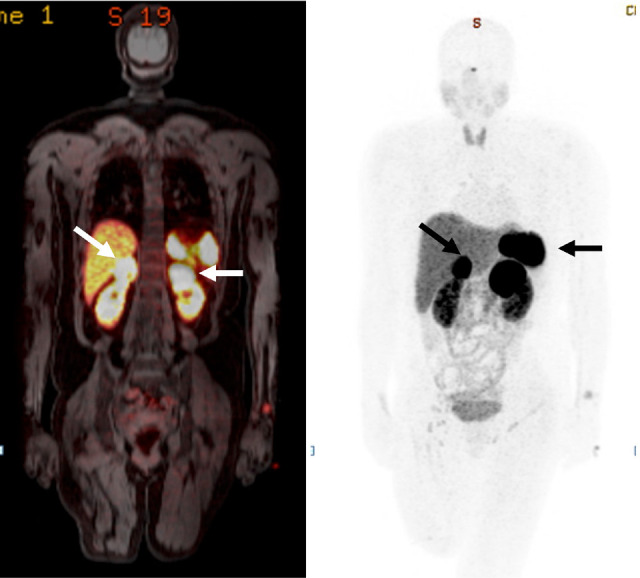
PET/MRI with somatostatin analog (68Ga-DOTATATE). Whole-body PET/MRI scan with Somatostatin analog (68Ga-DOTATATE) demonstrating heterogeneous radiopharmaceutical uptake at the left adrenal region (measuring 8.0 × 6.2 × 5.5 cm; AP × CC × LL), at the right adrenal region (measuring es 5.4 × 5.2 × 4.3 cm; AP × CC × LL), and in the intramedullary region of the left distal radius (indicated by the arrows).

Additional laboratory evaluation showed normal adrenal hormone levels (cortisol and aldosterone). Laboratory tests revealed prediabetes, severe hypercholesterolemia (LDL-C 270 mg/dL), markedly elevated TSH (9.29 µUI/mL [0.55-4.78 µUI/mL] and low FT4 0.86 ng/dL [0.93-1.7 ng/dL]) suggesting hypothyroidism-normal renal function and electrolytes, unremarkable complete blood count except mild leukocytosis, and borderline-high triglycerides. Calcium metabolism markers were within normal limits: PTH 33 pg/mL (10-65), vitamin D 21 ng/mL (>20), ionized calcium 4.92 mg/dL (4.49-5.29), and phosphorus 4.2 mg/dL (2.7-4.5).

Evaluation for hypertensive target organ damage showed on echocardiogram: aortic sinus 43 mm, left atrium 36 mm, left ventricular diastolic diameter 39 mm, systolic diameter 23 mm, interventricular septum thickness 18 mm, left ventricular wall thickness 10 mm, LV mass index 109 g/m^2^, and ejection fraction 73% (Teicholz method), with normal valves and pericardium. After this comprehensive evaluation, bilateral pheochromocytomas were confirmed. Blood pressure was controlled with prazosin and metoprolol; bilateral adrenalectomy was recommended.

### Surgical procedure and patient evolution

The patient first underwent left adrenalectomy, followed by right adrenalectomy seven days later. For the left pheochromocytoma, histology identified two adverse features(vascular and capsular invasion) each contributing 1 point, for a total pheochromocytoma of the adrenal scaled score (PASS) of 2. All other PASS criteria were absent, including large nests/diffuse growth (>10%) central or confluent necrosis, high cellularity, cellular monotony, spindle cell component, >3 mitotic figures per 10 high-power fields, atypical mitoses, adipose tissue invasion, marked nuclear pleomorphism, and nuclear hyperchromasia. Surgical margins were clear. For the right adrenal pheochromocytoma, PASS evaluation showed large nests/diffuse architecture (>10%) and high cellularity, each scoring 2 points, for a total PASS of 4/20. All other criteria were absent, including central or confluent necrosis, cellular monotony, spindle cell component, increased mitotic activity (>3/10 HPF), atypical mitoses, adipose tissue invasion, vascular invasion, capsular invasion, marked nuclear pleomorphism, and nuclear hyperchromasia.

Pathological exams confirmed the presence of the bilateral pheochromocytoma, with positivity Tumor cells were positive for Chromogranin, Synaptophysin, and S100 (in sustentacular cells in the left adrenal and focal areas in the right adrenal). Ki-67 proliferation index was 3-5% in the left and 1-2% in the right adrenal. Mosaicism for the pathogenic *H3F3A* c.103G>T/p.G35W variant was observed with bilateral pheochromocytomas and recurrent bone giant cell tumors. Both pheochromocytomas harbored this *H3F3A* variant, which was also found in a low allele frequency (<10%) in germline DNA. Germline DNA was analyzed using a targeted next-generation sequencing panel for pheochromocytoma susceptibility genes, and the results were negative for RET.

The patient’s variant (p.G35W) corresponds to the canonical *H3F3A* hotspot mutation historically referred to as p.G34W. There is no difference between the mutations themselves; the discrepancy arises solely from nomenclature. The updated HGVS guidelines recommend numbering based on the full-length reference sequence, including the initiator methionine, which shifts the amino acid position by +1. Under this updated system, the traditional p.G34W is now designated as p.G35W.

Four days after left adrenal removal, 24-hour urine catecholamines, plasma metanephrine, and 24-hour urine metanephrine were measured. Three days after right adrenal removal, a 24-hour urine sample was collected for catecholamine and metanephrine measurements (**Table 2**).

**Table 2:** 24-Hour urine catecholamines and plasma and urine metanephrine measurements after left and right adrenalectomy, respectively

Variables	Values	Reference values
Catecholamines and metanephrine levels after removal of left adrenal gland		
Plasma metanephrine (nmol/L)	0.8	< 0.5 nmol/L
Urine norepinephrine (µg/24 h)	279	14-80 µg/24 h
Urine epinephrine (µg/24 h)	Undetectable	0.5-20 µg/24 h
Urine dopamine (µg/24 h)	651	65-400 µg/24 h
Urine metanephrine (µg/24 h)	6.3	0.05-1.2 µg/24 h
Catecholamines and metanephrine levels after removal of right adrenal gland		
Urine norepinephrine (µg/24 h)	18	14-80 µg/24 h
Urine epinephrine (µg/24 h)	Undetectable	0.5-20 µg/24 h
Urine dopamine (µg/24 h)	232	65-400 µg/24 h
Urine metanephrine (µg/24 h)	0.27	0.05-1.2 µg/24 h

During surgery, part of the normal left adrenal parenchyma was preserved. For this reason, the patient does not require fludrocortisone replacement. Over the course of follow-up, she has maintained normal sodium and potassium levels, as well as renin at 26 µIU/mL (4.4-46.1 µIU/mL), with normal blood pressure and no orthostatic hypotension. The patient is receiving hydrocortisone supplementation and is taking atorvastatin 40 mg daily for cholesterol control. The most recent LDL-cholesterol value, measured in November 2024, was 104 mg/dL. Following adrenal resection, the white blood cell count decreased to 6,290 mm^3^. Plasma metanephrine and normetanephrine levels have remained stable and within normal limits during five years of follow-up (**Table 2**). The patient has not experienced recurrence of the giant cell tumor.

Plasma metanephrines during five years of follow-up were as follows: metanephrine <0.2 nmol/L in 2022, 0.2 nmol/L in 2023, <0.2 nmol/L in 2024; normetanephrine 0.5 nmol/L in 2022, 0.6 nmol/L in 2023, and 0.5 nmol/L in 2024. Post-adrenalectomy, the patient became normotensive off medication, and follow-up echocardiography showed regression of left ventricular hypertrophy.

## DISCUSSION

Pheochromocytomas and paragangliomas are rare catecholamine-secreting tumors of neural crest origin, arising from sympathetic lineage cells in the adrenal medulla and paraganglia, respectively. Notably, over 40% of pheochromocytomas are caused by dominant driver mutations in various susceptibility genes, affecting multiple biological pathways ^(3)^.

Pheochromocytomas significantly influence bone metabolism via catecholamine excess, which drives bone resorption and secondary osteoporosis, as shown in numerous studies ^(4-7)^. This is primarily attributed to sympathetic overstimulation, which disrupts bone microarchitecture, resulting in increased bone fragility, particularly in trabecular bone ^(6-9)^. Disabling bone pain has been reported in malignant pheochromocytoma ^(10)^. Pheochromocytomas are commonly associated with genetic syndromes, including MEN2, von Hippel-Lindau disease, and neurofibromatosis type 1 ^(1,11)^; however, these do not generally include primary bone tumors within their clinical spectrum. Therefore, the correlation between pheochromocytomas and primary bone tumors is not well established in the medical literature.

Recent studies investigating the molecular biology of PPGLs have identified a novel cancer syndrome linking PPGLs and GCT ^(3)^, attributed to a postzygotic G34W mutation in the histone 3.3 gene, H3F3A. These findings suggest that chromatin architecture changes in a substantial subset of PPGLs may be mediated by diverse mechanisms-including direct mutations of chromatin modifiers. In the context of histone 3.3 mutations, this misregulation manifests within a distinct clinical and developmental framework. The unique postzygotic H3.3 mutation pattern seen in our cases, previously unreported in other H3.3-mutant tumors, warrants further investigation into possible common progenitors for sympathetic lineage and bone tumors. This finding has important implications for assessing mutation transmission risk in affected individuals.

## CONCLUSION

This case provides compelling evidence for PPGL-GCTB syndrome caused by mosaic H3F3A mutations. Our findings underscore the importance of considering genetic testing in patients presenting with concurrent rare tumors, as this is essential for accurate diagnosis, genetic counseling, and long-term management.

## Data Availability

datasets related to this article will be available upon request to the corresponding author.
